# Spatial patterns of diabetes related health problems for vulnerable populations in Los Angeles

**DOI:** 10.1186/1476-072X-9-43

**Published:** 2010-08-27

**Authors:** Andrew J Curtis, Wei-An Andy Lee

**Affiliations:** 1 Department of American Studies and Ethnicity, College of Letters, Arts and Sciences, University of Southern California, Los Angeles, USA; 2 Keck School of Medicine, University of Southern California, Los Angeles, USA

## Abstract

**Background:**

Rates for Diabetes Mellitus continue to rise in most urban areas of the United States, with a disproportionate burden suffered by minorities and low income populations. This paper presents an approach that utilizes address level data to understand the geography of this disease by analyzing patients seeking diabetes care through an emergency department in a Los Angeles County hospital. The most vulnerable frequently use an emergency room as a common care access point, and such care is especially costly. A fine scale GIS analysis reveals hotspots of diabetes related health problems and provides output useful in a clinic setting. Indeed these results were used to support the work of a progressive diabetes clinic to guide management and intervention strategies.

**Results:**

Hotspots of diabetes related health problems, including neurological and kidney issues were mapped for vulnerable populations in a central section of Los Angeles County. The resulting spatial grid of rates and significance were overlaid with new patient residential addresses attending an area clinic. In this way neighbourhood diabetes health characteristics are added to each patient's individual health record. Of the 29 patients, 4 were within statistically significant hotspots for at least one of the conditions being investigated.

**Conclusions:**

Although exploratory in nature, this approach demonstrates a novel method to conduct GIS based investigations of urban diabetes while providing support to a progressive diabetes clinic looking for novel means of managing and intervention. In so doing, this analysis adds to a relatively small literature on fine scale GIS facilitated diabetes research. Similar data should be available for most hospitals, and with due consideration for preserving spatial confidentiality, analysis outputs such as those presented here should become more commonly employed in other investigations of chronic diseases.

## Background

Diabetes Mellitus is a considerable economic burden on the medical system in the United States. The incidence of diabetes has tripled from 5.6 million in 1980 to 17.4 million in 2007, and its prevalence has increased to 7.8% [1]. The total estimated cost from direct and indirect medical care in 2007 was an astounding $174 billion [1]. Unfortunately diabetes and its associated comorbidities such as hypertension and obesity are disproportionately experienced by minority communities and those living close to or below poverty [2]. This burden now has an additional cohort of concern in terms of an emerging epidemic of obesity and diabetes in children [3-5]. As a result there is a social and economic need to better understand and treat this disease, and to this end geography and geographic techniques can offer invaluable insights into both patterns and processes. Indeed geospatial approaches should be components of diabetes research due to the inherent spatial nature of many of the causative pathways, which include (but are not limited to) food insecurity, lack of safe activity spaces, and poor access to health services. This geographic approach can be further extended to include not just the residential space but also the daily sphere of activity [6].

The intersection between neighbourhoods and health has proven a fertile ground of investigation in social epidemiology. Although the complex interactions between people, health, space and place goes beyond the scope of this paper, it is enough to acknowledge that both contextual and compositional factors are likely to have an influence on neighbourhood variation on diabetes. For example access to healthy food options [3,7-11], is more complex than a simple proximity measure between food opportunities and a disadvantaged community, but also involves cultural choices, low income challenges (such as cost and food preparation time), accessibility (for example bus routes) and impediments (crime) [12-14]. Other moderating geographies can improve the overall living conditions in a neighbourhood, with examples being access to active living spaces, especially parks and recreation areas, and the greening of urban areas [4,5,11,15-18].

Understanding the geography of diabetes can also support the spatial prioritization of intervention or health care access [6]. Indeed, the utility of such spatial investigation may even extend beyond research and health care delivery to involve communities, helping them understand where and why disease occurs in their locales, and how the situation can be improved either through screening or lifestyle change with the additional benefit of bringing community members into the discussion through participation [19]. It is therefore a shame that relatively little fine scale spatial research on diabetes has been carried out (for exceptions see [20-22]). There have, however, been numerous investigations on the contributing environmental factors, though usually without the supporting evidence of actual diabetes data.

This paper illustrates how a commonly collected dataset (hospital patient records) can be used to provide geographic insight into diabetes and its debilitating associated conditions. This approach is worthwhile as although any one hospital will not provide exhaustive coverage for the entire area, the emergency department of a major county hospital is likely to treat a high proportion of the most vulnerable living in close proximity. The results of these neighbourhood scale analyses not only reveal patterns in the hospital's hinterland, but can also be used by local clinics tasked to target this most at-risk cohort. In so doing this paper shows how both research and practical application can be combined.

### Diabetes in Los Angeles

Within the state of California, Los Angeles County has the highest number of diabetes patients and as a result has the highest number of hospitalizations related to diabetes. Within this county, East Los Angeles has the largest urban Hispanic population in the United States with a prevalence of diabetes more than double that of Caucasians. In addition, the area has a high percentage of residents living in poverty, and again the prevalence of diabetes in this group is three times higher than those living at 200% of the federal poverty level [23].

Previous studies of diabetes in this minority community have shown that metabolic control and its maintenance is resource intensive and unsustainable [24,25]. In addition, unclear complex socioeconomic dimensions present barriers to the delivery of healthcare in this community. In the study by Rueles, social factors such as adherence to medications were highly predictive in determining patients who responded to a disease management program [26]. Unfortunately, variables that attempted to correlate metabolic control with demographic and behavioural characteristics were all non-statistically significant, for example education level, clinical visits, or country of origin [26]. As a result there is an ongoing need for the development of a methodology which can quantitatively capture other environmental variables impacting this "at risk" population that can then be targeted for intervention.

The Los Angeles County-USC Medical Center is a public hospital considered to be the safety net for health care access for Los Angeles County residents. It is the largest single provider of healthcare for the area's medically underserved community and the emergency department (ED) treats more than 300,000 visits a year. It is the population that seeks diabetes related care through this particular outlet that provides the focus of this paper. An analysis of people using the ED for primary diabetes care is important as it is suggestive of a vulnerable population to whom this is the only point of access to health care and whose actions pose the greatest cost burden to the system. This cohort is also likely to suffer the greatest chronic disease burden in general. In order to develop a neighbourhood scale analysis of where these diabetic sufferers live, and where spatial variation exists in terms of the health problems associated with diabetes, a fine spatial scale dataset is required. Using LAC+USC Medical Center data, this paper describes an analytical frame that can be transferred to other urban environments as datasets utilized here should be available in most urban settings. Although the specific situation described in this paper for Los Angeles should only be considered as an initial "exploratory" analysis, the following general research question can still be advanced:

Can hospital (Emergency Department) surveillance data reveal fine scale spatial variation in diabetes related conditions for a highly vulnerable population?

## Methods

Although the patient data used in this study does not provide a complete profile of the diabetes situation in Los Angeles County, the study area provides an invaluable insight into a geography dominated by those carrying the highest disease burden, most of whom would be expected to attend the Los Angeles County-USC Medical Center. Although there are likely to be pockets of diabetes sufferers living within this area who seek treatment elsewhere - especially those from more affluent situations, these should not affect the research question of the paper which is focused on the most socially vulnerable population suffering from diabetes. Indeed, an even more stringent geographic reduction of these data is used whereby only ED visits are analyzed, in other words investigating the most vulnerable and those causing the greatest "costs" to the health care system.

### Data

The Los Angeles County-USC Medical Center has an electronic medical record system which archives the physician designated International Classification of Disease code (ICD) for each patient encounter. It is assumed that the ICD code is accurately recorded in the emergency room data subset analyzed in this paper due to the experience of the medical staff working there, and the shared understanding that hospital record keeping should maintain a high quality [27]. In total 30158 records where a patient was labelled with diabetes were attained from the Los Angeles County-USC Medical Center. Patients in the ED are evaluated with a limited history and physical examination. They are then labelled with an ICD code for diabetes if they have a pre-existing history of diabetes or laboratory confirmation (eg. Random blood sugar of 200 or greater). Diabetes patients who do not provide a verbal history of diabetes to the ED staff or don't present with a condition that warrants laboratory investigation may not be appropriately labelled. Our study relies on the accuracy of this staff-dependant ICD coding for diabetes. From this total 20257 records were confidently address matched, with non matches falling into the usual categories of spelling mistakes, inappropriate field entries, and non-spatially specific "homeless" records. As the purpose of this paper was to establish spatial patterns of diabetes characteristics, and not establish a complete record of the disease, only those addresses matched with an extremely high degree of confidence were retained. For example, imputation approaches were not used (for example using zip code centroids) to compensate for spatial entry uncertainties [28]. As there is no expected geographic bias in the non-matched addresses (apart from the category of "homeless"), and as this dataset would never capture the entire diabetes situation for the area, the successfully matched addresses should be considered as a suitable sample for the proposed analysis.

These records contain basic social characteristics, including age, sex, and race. In addition, diagnoses and place of contact are also recorded. As the data set contains all diabetes related visits during 2009, many individuals appear as multiple records. Therefore, using the machine run identifier (the database method employed by the hospital to identify a person's record), a subset of 8875 unique individuals were extracted. From these, a further subset was selected and kept for analysis of those patients who had used "emergency department visit for the evaluation and management" for their diabetes related problem. This subset of 3522 records would be used as the base layer for the subsequent spatial investigations of diabetes.

When using a secondary dataset of this type, for any analysis involving the calculation of disease rates a suitable "denominator" has to be determined. As one purpose of this paper is to show how spatial patterns of associated health problems occur within one vulnerable cohort, the total number of unique individuals being studied (3522 patients) is used as the denominator. The benefit of this approach is that the chance of a bias propagating because of the dataset being analyzed is minimized. If certain neighbourhoods generate a disproportionate number of ED treatment seeking individuals, then these will also likely present as hotspots for the related conditions being analyzed. By using ED attendees as the denominator this effect is controlled for and the analysis reveals spatial patterns from within this cohort. Therefore the hotspots that are identified are only of the population being studied (ie those who have diabetes and receive treatment through the ED). Using this subset as a denominator surface, a series of smoothed disease rate surfaces are calculated using DMAP spatial filtering software [29] for the following diagnostic categories (ICD listed):

1. Uncontrolled Blood sugars including both type 1 diabetes (250.01) and type 2 diabetes (250.02) in this ED population.

2. End organ damage related to uncontrolled diabetes in the following:

250.4 = kidney issues with diabetes

250.6 = neurologic issues related with diabetes

250.8 and 250.9 being combined as a category for non-specified "other" complications of diabetes

For a patient to be considered a numerator for any of the specified ICD analyses, he/she had to have one of the previously listed codes appearing in the primary (first) diagnostic column - suggesting this symptom was the main cause for the ED visit. Despite the differences in the underlying mechanism of disease between type 1 and type 2 diabetes, this underserved, predominantly minority population have the same social barriers that limit their adherence to medical regimens and therefore lead them to the ED for primary or emergent care. In addition, we chose to evaluate complications that occur in both types of diabetes, eg. hyperglycemia, renal, and neurologic disease.

Although the main objective of this paper was to show the potential for using ED data to reveal neighbourhood patterns in diabetes, a secondary aspect of this research is to combine both an academic investigation with a "real world" practical application. A collaboration has been established between the Department of American Studies and Ethnicity at the University of Southern California and an outpatient Diabetes and Metabolism Clinic (DMC) staffed by an endocrinologist and two nurses associated with the Los Angeles County-USC medical center. This clinic implements a Diabetes Management Program with the aim of the global risk reduction of metabolic parameters (blood sugar, blood pressure, and lipids) utilizing management guidelines from the American Diabetes Association and American Heart Association in a culturally appropriate format. Diabetic patients are referred from their primary care physicians when their glycemic control is difficult to manage. This population is predominantly living below the federally defined poverty level and has no established health care coverage beyond the free access provided to them from the Los Angeles County Healthcare System. Due to the close proximity of this clinic to the Los Angeles County-USC Medical Center ED, the DMC predominantly serves the same underserved community. A selection of patients (29 in total) attending this new clinic are overlaid onto the hotspot surfaces in order to provide an assessment of the diabetes situation surrounding their home. In other words, the actual residences of the patients are placed on the maps of diabetes related health problems. This approach of combining "new" patients with a background disease surface can also provide a mechanism for assessing the success of new clinic strategies by comparing changes in patient outcomes to their ecological situation. In addition, for the purposes of this paper, these patients also provide a set of sample locations drawn from the same population against which the background diabetes disease surface can be described.

### Spatial Analysis

There are several spatial models which could have been applied to reveal neighbourhood scale "hotspot" patterns [30-34]. For this paper DMAP software [35] was chosen due to its established success of analyzing fine scale health information, including birth outcome and cancer data [29,36]. The basic premise of DMAP is a grid is overlaid on the study area, a circular filter is placed over every grid node and a rate is calculated with the numerator being comprised of patient addresses for a certain ICD, and the denominator being all patient addresses diagnosed as having diabetes who made visits to the ED. As long as the filter radius is greater than half the distance between nodes, the calculated rate for each filter will involve points used in multiple rate calculations resulting in a smoothed rate surface that is not truncated by political boundaries. In general, the larger the radius of the filter, the more points are included in the rate calculation and the smoother (and less volatile) the final surface tends to be. The output from DMAP consists of a grid with a rate calculation attached to each node which can be imported into a GIS and mapped as either an interpolated surface, or as contours.

**Figure 1:**
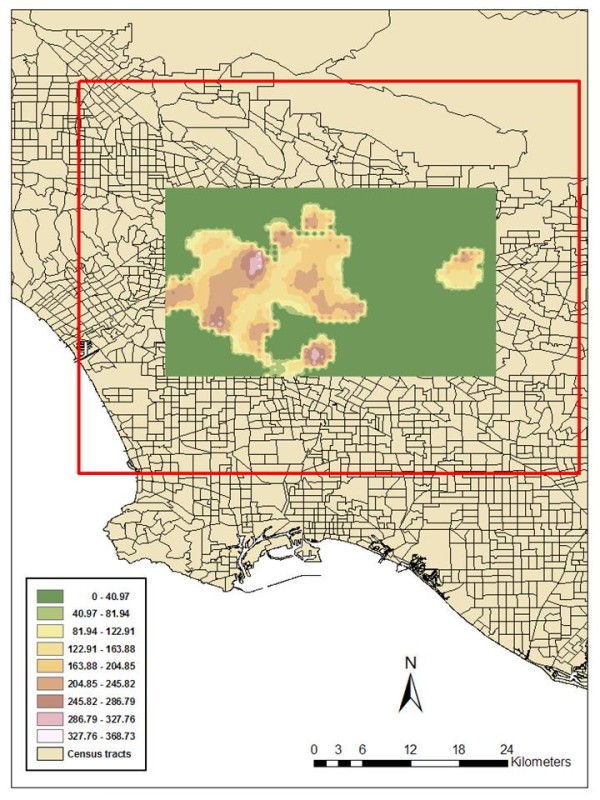
**A rate surface for diabetic patients attending the Los Angeles County-USC Medical Center**. Figure 1 displays the geographic extent of the Los Angeles County study area displayed as an Inverse Distance Weighted (IDW) smoothed grid of all those patients who made ED visits whose primary diagnosis was diabetes related. This is expressed as a rate (per 1000 ED visits), therefore, the highest rate is almost 370/1000. This means, if 1000 patients attended the ED with diabetes, the primary diagnosis was directly related to a health problem resulting from that disease (in effect one of the ICD codes investigated in this paper) - this being their reason to seek medical attention. The outer red box identifies the larger study area which was included to assess the stability effect of spatial scale.

For this paper a geographic subset of Los Angeles County (30 km by 15 km) was selected as the study area based on the output of a density analysis of the total (20257) records from the Los Angeles County-USC Medical Center. This geographic extent can be seen in Figure 1. In order to compare scale effects that might occur through such a boundary selection, a secondary analysis was also performed for a larger geographic area. Both extents were covered by a grid with a resolution of 0.5 km, and rates were calculated for a filter extending to 1 km from each node. Multiple band widths for the filters were tried, and the one chosen (twice the distance between nodes) corresponds to similar appropriate ratios found by the authors in other applications of this technique. In order to reduce the error associated with the law of small numbers, only filters with at least 30 denominator values were recorded, all other nodes on the map being removed. The output of rates attached to each grid node was imported to Arc Map GIS, where interpolated surfaces for each ICD rate were calculated. These interpolated grids were also contoured so that they could easily be overlaid on the underlying geography to aid in interpretation and spatial comparisons.

A test of statistical significance was also applied to the rate surface using a Monte Carlo simulation within DMAP. Each denominator address was given the chance of becoming a numerator ICD based on the proportion of that condition found in the actual population under investigation. A simulated surface was calculated using these probabilities - in effect the same overall ICD rate was maintained for the study area, but the locations of the numerators changed. By repeating this simulation for 1000 runs, a comparison distribution is created. The actual rate for any one node can then be compared against this simulated distribution to determine how frequently the "real" rate exceeded the "simulated" rate. Therefore, if the actual disease rate was higher in any neighbourhood than in 950 of the 1000 simulations, we can be 95% confident of determining this location to be a hotspot.

The secondary aspect of this paper was to illustrate how the analytical insight can also be used to support near-real time diabetes control. To this end, patient addresses from a diabetes clinic were overlaid onto the DMAP output maps of rates and significance. This meant that each of the new clinic's patients were matched to their closest node, with all the attributes of that node (the statistical significance values for each ICD being investigated, and the separating distance between the address and the node) being attached to the patient's address.

## Results

Of the 3522 patients with diabetes, 545 had made multiple visits to the ED over the course of 2009. For example, the mode number of visits was 2 (400 patients), 94 made 3 and 24 made 4 ED visits respectively. This does not mean these were the only medical visits made by each patient, rather a count of only those to the ED which are analyzed here.

Figure 1 is the geographic extent of the Los Angeles County study area displayed as an Inverse Distance Weighted (IDW) smoothed grid as output from DMAP with inputs of patients who made ED visits whose primary diagnosis was diabetes related.

**Figure 2:**
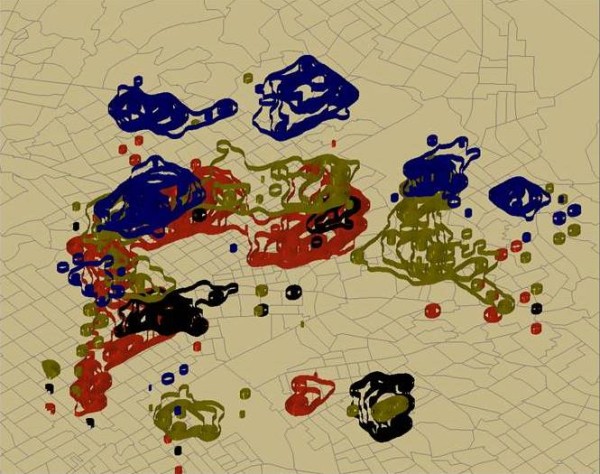
**Floated rate hotspots of primary diagnosed diabetes related conditions**. Figure 2 displays four contoured surfaces "floated" above a census tract boundary map in Arc Scene. By stacking these contoured surfaces the spatial pattern can be more easily investigated, while spatial confidentiality control is preserved for publication purposes. Green shows both type 1 and type 2 diabetes, blue represents kidney issues with diabetes, black shows neurologic issues related with diabetes, and red complications of diabetes in the "other" categories.

Similar IDW grids were calculated for Uncontrolled Blood sugars including both type 1 diabetes (250.01) and type 2 diabetes (250.02), 250.4 = kidney issues with diabetes, 250.6 = neurologic issues related to diabetes, and 250.8 and 250.9 combined as a category for a primary diagnosis of "other" complications arising from diabetes. These grids were also contoured using Arc Map GIS 9.3 and displayed within the 3 D environment of Arc Scene to allow comparisons between layers but preserve confidentiality in publications (Figure 2).

**Figure 3:**
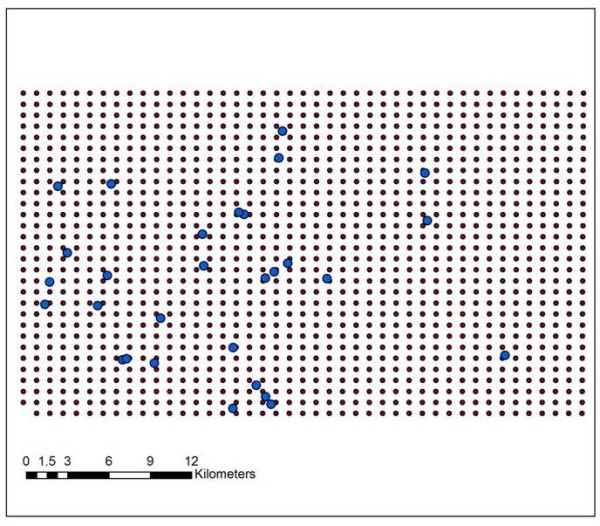
**Overlaying "new" diabetes patient addresses on the background neighbourhood rate grid**. In order to make further comparison between the new clinic's patients and the neighbourhood disease burden, a test of statistical significance was calculated for each node. Each of the 29 patients home address was assigned to the closest node, these being displayed in Figure 3 which shows both the grid and the residential locations. All other geographic information is removed to eliminate the possibility of reverse engineering patient addresses [37].

For comparison purposes, 29 patients attending a new diabetes and metabolism clinic were overlaid onto these maps and each of their neighbourhood disease rates recorded and displayed in Table 1. In order to make further comparison between the new clinic's patients and the neighbourhood disease burden, DMAP was also used to create maps of statistical significance for every ICD being investigated. These were overlaid with the clinic's patient's residences (Figure 3). Results can be seen in Table 2 where each patient along with his/her clinical diagnosed diabetes measures is matched to the closest node. These include the body mass index (BMI) expressed with the ratio of kilogram to meter^2 ^which is a standardized method to measure weight and is used to assess the level of obesity, Haemoglobin A1C(HBA1C) a measure of the average plasma glucose over a long duration and generally used to gauge glycemic control in diabetes patients, low density lipoprotein(LDL) and triglycerides(TG) which are plasma lipid variables used to monitor lipid control. The significance value for each of the four ICD values can be interpreted in the following way, patient 14 lives close to a node (within 224 meters) where the actual rate for a primary diagnosis of neural conditions was higher than in 997 of the simulation runs. In other words we are 97% confident that there is a significant cluster at this location. In order to help with interpretation, those nodes exceeding a 90% confidence level are in bold, while those exceeding 80% (but less than 90%) are in italics. The distance column (in meters) ranges from 74 to 532 meters - meaning this is how close the new patient's address is to the node in the rate and significance grid. This justifies the comparisons as all distances were within the size of the filter radius.

**Table 1:** Comparing "new" diabetes patient addresses to their closest background neighbourhood rate node

Patient	D1&2	neuro	renal	other	dx	D1&2L	neuro L	renal L	other L	dxL
0	0	0	0	0	35.56	0	0	0	0.38	35.59
1	**28.89**	5.46	11.13	**27.45**	**229.47**	**34.11**	6.46	9.71	**29.54**	**265.42**
2	3.46	11.26	0	0.56	**115.82**	5.74	4.62	0	0.9	93.04
3	13.78	2.36	11.41	3.52	**127.99**	14.72	1.48	13.24	12.35	**156.5**
4	3.87	13.56	7.67	1.88	**195.79**	4.21	13.69	7.54	2	**193.58**
5	0	0	0	0	0	0	0	0	0	0
6	14.15	1.41	12.75	7.21	**162.69**	22.44	0.37	22.08	5.4	**221.37**
7	0	0	0.78	0	3.13	0	0	0.55	0.55	2.73
8	**35.56**	0	2.87	1.72	**151.45**	**33.46**	0	1.96	0.6	**140.37**
9	15.31	14.35	20.14	**50.47**	**219.27**	13.55	17.94	14.22	**51.28**	**209.29**
10	0	0	0	0	0	0	0	0	0	0
11	0.29	0	1.4	1.04	8.87	2.2	0	2.32	3.91	19.16
12	4.3	15.44	0.41	19.08	**177.6**	3.46	14.1	0.17	17.12	**193.58**
13	**34.46**	0	2.32	0.2	**142.32**	**29.68**	0	4.93	0.2	**145.33**
14	3.15	0	**41.98**	19.98	**236.46**	1.54	0	**45.97**	22.64	**233.78**
15	0	0	0	0	0	0	0	0	0	0
16	0	9.81	2.61	8.43	**182.39**	12.81	10.43	2.37	8.47	**198.44**
17	1.08	0	**45.57**	3.69	**184.07**	0.56	0	**50.32**	2.44	**172.26**
18	18.52	6.37	17.71	**27.88**	**153.29**	14.3	13.61	13.3	**28.36**	**141.4**
19	1.03	**40.23**	0	14.3	**155.62**	0.94	**39.62**	0	13.37	**160.7**
20	0.32	0	**46.73**	21.16	**174.07**	0.62	0	**49.88**	21.2	**178.55**
21	18.93	0	9.43	1.46	**135.5**	21.64	0	10.43	2.05	**156.81**
22	5.51	2.07	3.44	2.07	46.69	4.5	1.47	3.03	3.03	38.46
23	0	12.91	1.47	1.95	**186.82**	10.6	12.53	2.78	4.74	**202.97**
24	4.1	0.57	27	14.21	**133.7**	4.17	0.56	**28.84**	21.17	**145.65**
25	0	0	0.31	22.99	**232.07**	0	0	7.54	5.83	**239.24**
26	12.98	0	0	0	55.39	5.12	0	0.34	0	21.45
27	0	0	0	0	0	0	0	0	0	0
28	9.19	18.57	0.88	2.04	**153.52**	9.8	18.14	0.73	5.07	**157.25**

This table can be interpreted in the following way. Patient 20 lives in a neighbourhood (defined by DMAP output) where of 1000 diabetic patients using the ED, 174 had a diabetes related condition as their primary reason for the visit, and more specifically where 46 per 1000 patients suffer primarily from renal problems. For ease of interpretation, any rate for the four conditions under investigation exceeding 25/1000 are in bold. The "dx" value is taken from the surface generated in Figure 1. In addition, for the sake of completion, the same rate results are reported for the larger boundary study area (the red box in Figure 1).

**Table 2:** Comparing "new" diabetes patient health markers to statistically significant neighbourhood hotspots of diabetes related conditions

	BMI	HBA1C	LDL	TG	Ethnicity	Diab	neural	other	renal	Dist
0	25	11	114	190	H	0	0.72	0	0	418
1	29	9	108	80	H	0	0	0	0	335
2	28	17	139	109	H	0.5	0	0	0.63	414
3	23	10	58	105	H	0.53	0	0.57	0.66	532
4	32	10	56	170	H	0	0	0	0	385
5	30	8	72	58	H	0	0	0	0	199
6	23	15	97	220	H	0	0	0.58	0.67	383
7	42	15	34	195	H	0	0	0	0	256
8	24	5	104	144	H	0	0	0	0	186
9	35	11	0	0	H	0.27	0	0.67	0.78	387
10	30	9	65	47	H	0	0	0	0	316
11	33	8	87	45	H	0.75	0	0.42	0.5	379
12					AF	0	0	0.49	0	74
13	43	13	142	304	H	0.42	0.28	0.18	0	243
14	33	12	87	94	H	0	**0.97**	0.33	0	224
15	39	9	67	155	H	0.27	0.41	**0.99**	0	471
16	33	8	86	103	H	0.15	0.67	0	0	466
17	27	12	0	0	H	0.06	0.48	0	0	189
18	25	9	152	176	H	0	0.5	0	0.45	344
19	42	12	51	87	H	0	0	0	**0.96**	433
20					AS	0	***0.82***	0.3	0	410
21	30	11	86	72	H	0	0	0	0	457
22	27	10	69	253	H	***0.81***	0	0	0	215
23	34	10	101	69	H	***0.82***	0	0	0	355
24	32	9	113	536	H	0.5	0	0	0	474
25	33	10	0	415	H	0	0	0.64	**0.98**	362
26	65	12	75	95	H	0	0	0	0	219
27	30	10	127	201	H	0	0	0.51	**0.92**	174
28	31	10	116	97	H	0.51	0	0	0	493

Each patient has been linked to his/her neighbourhood rate. This table displays both the patients diabetes related health markers and whether his/her neighbourhood is statistically significant for one of the investigated ICD conditions. Where BMI = body mass index, HBA1C is HemoglobinA1C, LDL is low density lipoprotein, and TG is triglycerides. Also H = Hispanic, AA = African American and AS = Asian (data for both withheld for confidentiality reasons).

## Discussion

Diabetes rates, along with other comorbidities are on the rise in vulnerable populations of US cities. The geographic complexity surrounding these diseases, both in terms of negative and positive spaces, necessitates the need for more GIS facilitated investigations. Understanding these geographies can both advance our understanding of the disease while also spatially prioritizing health care delivery and intervention. This paper has made a first exploratory investigation of diabetes in Los Angeles for 2009, determining the spatial patterns of diabetes conditions as identified by ICD code diagnoses during ED visits. These results should be viewed with caution as the data being analyzed are spatially, temporally, and diagnostically complex. Indeed, many patients made more than a single visit for medical treatment, but only their ED visits were included in this analysis. The choice of only using ED data was made for two reasons, firstly to use a degree of standardization in the analysis, and secondly provide information for the ongoing dialogue as to how costly this disease is to society by concentrating on the most expensive of medical interactions.

**Figure 4:**
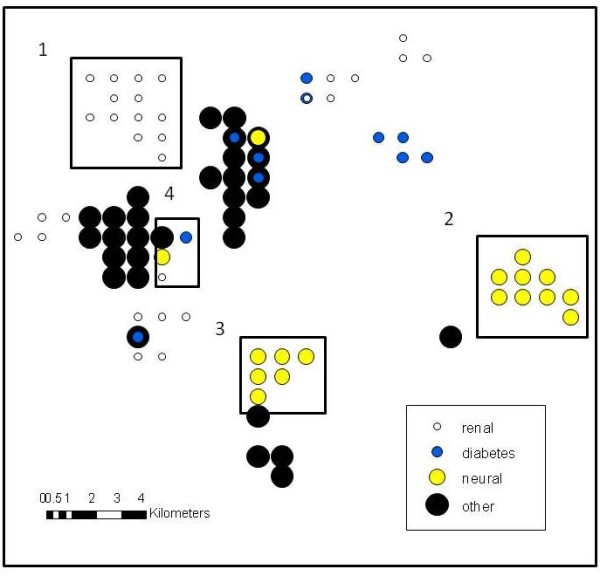
**Statistically significant nodes of diabetes related health conditions**. Figure 4 displays significant nodes reaching a 90% level of confidence as determined by DMAP analysis for four different diabetes related health problems.

One objective of the paper was to show how data that is normally collected by hospitals could be used to gain neighbourhood insight into local area chronic diseases. Although this is still an exploratory analysis, these results reveal interesting neighbourhood scale questions worthy of further investigation. For example, consider Figure 4 which shows only significant nodes reaching a 90% level of confidence. Four areas that would make interesting follow-up investigations would be "1" for renal, "2" and "3" for neural, and "4" for a combination of all ICD codes investigated. Although the smoothing aspect of this analysis technique might account for some neighbourhood grouping of significant nodes, the number of nodes contained in "1" and "2" especially warrants further study.

Subsequent analyses would extend spatial investigations to other diagnoses (for example amputations), include all visits to medical outlets, and even add a temporal component by comparing records across multiple visits, and for multiple years [27]. In so doing a more complete disease environment for this section of Los Angeles would be developed.

The complexity involved in these data also lend themselves to other forms of spatial investigation - for example using the exploratory capabilities of the GIS to "drill down" to neighbourhoods of interest to see not only where clusters of diabetes related conditions occur, but also how features on the landscape, such as housing type, service provision, even crime locations intersect with these findings. Indeed, these data surfaces would provide an interesting addition to many other urban investigations such as assessing disaster related social vulnerability (an important consideration in Los Angeles County). From a modelling perspective these data are also suitable for research at multiple spatial scales, meaning how would different aggregations of these data affect the location of hotspots. This would help shed further light on the impact of the modifiable area unit problem effect in these types of analyses.

The second purpose of this paper presented the utility of these data to support day-to-day clinic operations in terms of assessing neighbourhood risk factors. The approach described here can provide an invaluable neighbourhood scale insight where none is usually available for a clinic working with diabetes patients. Indeed, using hospital data such as these can help identify neighbourhood diabetes patterns, which in turn can be used to suggest contributing local environmental factors - leading to the targeting of intervention strategies. Specific patient level investigations into daily activity spaces would be more insightful both in terms of understanding where life-style improvements can be made, as well as finding social network solutions to target interventions [6]. However, such detailed investigations are often not practical for a financial and resource limited clinic and the results presented in this paper provide a reasonable alternative in making the first step to geographically profiling this disease. For example, of the 29 patients, five reside in statistically significant (at a 90% confidence level) hotspots for one of the ICDs being investigated (three for renal, one for neural and one for other diabetes related conditions). By using these results, along with the rates presented in Table 1, useful insight about the background extent of the disease has been provided to the Diabetes and Metabolism Clinic, they being the primary beneficiary of this initial analysis. However, the purpose of this paper was not to just provide a case study, but rather present a methodological frame that is transferable to any urban environment with a primary hospital serving a similar population, and willing to collaborate with address level data.

## Competing interests

The authors declare that they have no competing interests.

## Authors' contributions

AJC conceived of this study, performed all GIS and spatial analyses, and drafted the manuscript. WAL provided all primary and secondary data, helped conceive the study, and helped to draft the manuscript. All authors read and approved the final manuscript.
